# Sleep Quality in Chilean Professional Soccer Players

**DOI:** 10.3390/ijerph18115866

**Published:** 2021-05-29

**Authors:** Carlos Jorquera-Aguilera, Guillermo Barahona-Fuentes, María José Pérez Peña, María Mercedes Yeomans Cabrera, Álvaro Huerta Ojeda

**Affiliations:** 1 Facultad de Ciencias, Escuela de Nutrición y Dietética, Universidad Mayor, Santiago 8580745, Chile; carlos.jorquera@mayor.cl; 2 Grupo de Investigación en Salud, Actividad Física y Deporte ISAFYD, Universidad de Las Américas, sede Viña del Mar 2531098, Chile; danielbarahonaf@gmail.com; 3 Facultad de Ciencias, Escuela de Nutrición y Dietética, Magíster en Nutrición para la Actividad Física y Deporte, Universidad Mayor, Santiago 8580745, Chile; mariajoseperezp@gmail.com; 4 Facultad de Educación, Universidad de Las Américas, sede Viña del Mar 2531098, Chile; mmyeomans@outlook.com

**Keywords:** sleep quality index, physical performance, professional soccer players

## Abstract

Recent research has shown that good sleep quality has a positive effect on physical performance. However, sleep quality in Chilean professional soccer players is unknown. The purpose of this study was to determine sleep quality in Chilean professional soccer players. It was a cross-sectional, explanatory study with observable variables. The sample consisted of 94 Chilean male soccer players belonging to four professional clubs. The main variable was the Sleep Quality Index, evaluated through the Pittsburgh questionnaire (Spanish version). After estimating sleep quality individually, the four professional soccer clubs’ comparison was performed through a one-factor ANOVA. The Pearson test was used to relate the questionnaire variables; the significance level was *p* < 0.05. In the global analysis of the Pittsburgh Sleep Quality Index, a value of 4.75 ± 2.29 on a scale of 0–21 was observed, with no significant differences between the clubs evaluated (*p* > 0.05). Based on the results obtained, Chilean male professional soccer players present good sleep quality. However, the high values of “sleep latency” and “sleep disturbances” are indicators that should be worked on by the multidisciplinary team of each professional club. They should develop strategies to improve sleep hygiene, encourage good sleep, and fall asleep efficiently.

## 1. Introduction

Sleeping is a fundamental biological and physiological need of the human being. In fact, good sleep quality is a relevant factor for correct organic functioning; sleeping is regulated by the hypothalamus and cannot be omitted without experiencing harmful consequences for people [1] since, among other actions, it allows regulating and restoring psychological and physical functions [2]. Likewise, due to the brain’s multiple electrical activities during sleep, sleeping is considered a state of dynamic consciousness [3,4]. Specifically, the brain activity developed during the hours of sleep modifies the organism’s functioning; body temperature, specific hormone levels, blood pressure, and respiratory frequency might change [5]. Consequently, these organic changes, the product of brain activity during sleep, intervene in energy restoration processes, learning, memory, and cognition [1,6], favoring a state of recovery of people [7]. However, despite the existing evidence, a considerable number of people sleep fewer hours than necessary (sleep restriction) and have a low sleep quality [8].

Both the experience reported by patients [9] and scientific evidence [10] have established that people should sleep between 7 and 9 h a day for good sleep quality. On the contrary, generating a sleep restriction below the recommended time range (7 to 9 h) could generate a poor sleep quality [11], affecting the health of both common people [12,13] and elite athletes [14]. In recent years, it has been shown that poor sleep quality is associated with alterations in cognitive function, mood changes [15], endocrine system dysfunction [16], and depression of the immune system [17], among others [18]. Similarly, Spiegel et al. [19] showed that a four-hour daily sleep restriction for two consecutive nights reduced leptin levels and increased ghrelin levels in healthy adults; the researchers also associated these hormonal changes with an increase in hunger and appetite [19]. The latter could explain the modifications in body mass and overweight in people with sleep restrictions [20]. This background allows us to observe that sleep restriction, due to environmental factors, lifestyles, or diseases [21], affects the entire population [20,22,23,24].

There is evidence of deficiencies in the quality and quantity of sleep in elite athletes [14]. Specifically, Mah et al. [25] investigated the effects of sleep extension on sports performance, reaction time, mood, and daytime sleepiness in college basketball players, demonstrating that optimal sleep is probably beneficial for achieving maximum sports performance. Conversely, it has been established that poor sleep quality affects anxiety levels [26]; the latter condition is considered a negative emotion that decreases athletic performance [27], as it affects athletes’ perception before a competition [26]. To mitigate sleep restrictions in elite athletes, generated by uncontrolled variables such as anxiety, and because high sleep quality is a critical component for sports performance [28], multidisciplinary teams have implemented sleep education and sleep hygiene methodologies [29,30,31]. However, before implementing sleep hygiene programs, sleep habits and sleep quality should be evaluated in different populations [32,33].

Sleep habits and sleep quality are well documented in the adult population [32] and some populations with special needs [20]. In this sense, and considering that elite athletes have been described as populations with special requirements and needs [34], sleep quality and hygiene have been a focus of attention [29,30,31]. Another critical factor within sleep hygiene, which has also been extensively studied, is the circadian cycle [35,36]; indeed, there is strong evidence linking disrupted sleep and circadian misalignment with weight gain, obesity, and adverse effects on individuals’ metabolic health [35]. Therefore, as evidenced, adequate sleep at an appropriate time is essential for biological systems [36] and sports performance [28]. In this context, soccer is a social phenomenon that ranges from recreational to professional practice [37]. Likewise, there is evidence that soccer practice generates cardiovascular and metabolic benefits, helping to prevent diseases such as diabetes and hypertension [38]. Therefore, and considering that a large part of the population practices this sport, programs have been implemented to increase physical capacities [39], injury prevention [37] and good practices during rest periods [31]. Consequently, evaluating parameters such as sleep quality in athletes, specifically in Chilean professional soccer players, is a priority since good sleep hygiene ensures energy restoration, improves learning, memory, and cognition [1,6].

Despite the existing evidence and bearing in mind that many athletes present deficiencies in sleep quality and insomnia [40], studies in Chilean elite athletes are scarce [33]. In this context, only sleep quality, somnolence, and insomnia have been documented on Chilean elite Paralympic athletes [33]. Based on this background, and considering that sleep quality is a variable that conditions optimal sports performance [28], this study’s main purpose was to determine sleep quality in Chilean professional soccer players. Secondary objectives were a) to quantify the number of sleep hours of Chilean professional soccer players; and b) to demonstrate, if there are, differences in sleep quality between different Chilean professional soccer clubs.

## 2. Materials and Methods

### 2.1. Research Design

Explanatory, cross-sectional research, with observable variables [41].

### 2.2. Participants

Ninety-four (94) Chilean male soccer players belonging to four professional clubs volunteered to participate in this study (*n* = 94; age = 25.6 ± 5.3). The evaluated clubs were Unión Española (*n* = 20), Deportes Antofagasta (*n* = 18), Palestino (*n* = 32), and Universidad de Chile (*n* = 24). The type of sampling was non-probabilistic by convenience. To be included in the study, participants had to be men, practice professional soccer for a minimum of four years, and train regularly between four to six times per week. The latter excluded participants with pathologies, musculoskeletal injuries, or a training frequency of fewer than four times a week. Before answering the questionnaires, and with all doubts resolved, the participants signed the informed consent form.

### 2.3. Procedure

After reading and signing the informed consent form, all participants were asked to answer the Spanish version [42] of the Pittsburgh Sleep Quality Index (PSQI) questionnaire [43]. Both the informed consent and the questionnaire were applied at the training site and before beginning any type of physical exercise.

### 2.4. Materials

PSQI. This questionnaire was created by Buysee et al. [44] and adapted to Spanish by Jiménez-Genchi et al. [42]. The instrument’s objective is to self-assess the quality and subjective disturbance of sleep during a time interval of one month. Seven components assess this sleep quality: subjective sleep quality, sleep latency, sleep duration, sleep efficiency, sleep disturbances, the use of sleep medications, and daytime dysfunction [42]. Although the PSQI does not inquire about the specificity of the medication used to fall asleep, it is crucial to record them as psychiatric [45] and non-psychiatric medication qualitatively [46]. The questionnaire has two parts; the first consists of four items (each item with an open question): 1. During the past month, when have you usually gone to bed at night? 2. During the past month, how long (in minutes) has it usually taken you to fall asleep each night? 3. During the past month, when have you usually gotten up in the morning?, and 4. During the past month, how many hours of actual sleep did you get at night? The second part has six items with a total of 20 questions (each of these questions has four alternatives: 5. During the past month, how often have you had trouble sleeping because you... 6. During the past month, how would you rate your sleep quality overall? 7. During the past month, how often have you taken medicine (prescribed or “over the counter”) to help you sleep? 8. During the past month, how often have you had trouble staying awake while driving, eating meals, or engaging in social activity? 9. During the past month, how much of a problem has it been for you to keep up enough enthusiasm to get things done? and 10. Do you have a bed partner or roommate? The participant must only answer up to item nine of the questionnaire since item 10, which consists of five questions, must be answered by the “bed partner.” Only the self-assessed questions are included in the scoring. The 19 self-assessed items are combined for the scores for the seven “components.” Each of them has a range of 0–3 points. In all cases, a score of “0” indicates no difficulty, while a score of “3” indicates severe difficulty. The scores of the seven components are then summed to obtain an “overall” score, with a point range 0–21, “0” indicating no difficulty, and “21” indicating severe difficulty in all areas [42]. It should be noted that, for this study, a score ≤5.0 was considered good sleep quality [47].

### 2.5. Statistical Analysis

The results obtained in the PSQI questionnaire are presented with their mean data and respective standard deviation (SD). The data were subjected to the Kolmogorov and Smirnov normality test. Comparison of the variables between the different professional soccer clubs was performed through a one-factor ANOVA. We used the Student’s t-test to compare participants who scored ≤5.0 with those who scored over 5 points on the Pittsburgh questionnaire on the sleep variables. On the other hand, the effect size was calculated using Cohen’s d-test. The latter analysis considers an insignificant (d < 0.2), small (d = 0.2–0.6), moderate (d = 0.6–1.2), large (d = 1.2–2.0), or very large (d > 2.0) effect. To relate the different variables of the questionnaire, we used the Pearson test. This correlation coefficient was interpreted through classifications described by Mukaka [48], in which 0.9–1.0 corresponds to a very high correlation; 0.7–0.9 high correlation; 0.7–0.5 moderate correlation; 0.3–0.5 low correlation; and 0.0–0.3 very low correlation. Statistical analysis was performed with SPSS software. The significance level for all data was *p* < 0.05.

## 3. Results

The analysis showed that the 94 Chilean male professional soccer players sleep an average of 7.27 ± 0.92 h per day. In addition, it was observed that 53% sleep less than 7 h a day. Simultaneously, 45% of the soccer players responded that they take between 20 and 40 min to fall asleep, 19% take between 50 and 120 min to fall asleep, and 36% manage to fall asleep in less than 10 min. A relevant data within this analysis was the bedtime of the soccer players (this time corresponds to the sum of the time to fall asleep and the effective sleep time), reaching 8.20 ± 1.00 h. At the end of the analysis, it was also possible to observe a significant difference in bedtime between the different soccer clubs evaluated (*p* < 0.05). The data and their significance are reported in Table 1.

According to the scores obtained in the PSQI questionnaire [42], it was observed that two of the seven components (component 2: sleep latency and component 5: sleep disturbances) had a value over one on a scale of 0–3. This observation was made in both analysis by clubs and as a whole. In the global analysis of the Pittsburgh Sleep Quality Index, it was observed a value of 4.75 ± 2.29 on the 0–21 scale and no significant differences between the clubs evaluated (*p* > 0.05). When comparing the soccer players who scored ≤5.0 with those who scored over five points on the Pittsburgh questionnaire in the sleep variables, we could observe significant differences in the time to fall asleep (*p* < 0.001) and in 6 of 7 components of the Pittsburgh questionnaire (*p* < 0.05). The data are reported in Table 2 and Table 3.

Considering question 5 of the PSQI questionnaire, “During the past month, how often have you had trouble sleeping...?”, it was observed that 62.8% of the participants reported difficulty falling asleep at some point during the last month. Simultaneously, 78.7% answered they woke up during the night or in the early morning, and 72.3% woke up to use the bathroom. The details of each of the responses to question 5 of the questionnaire are reported in Table 4.

Pearson’s test showed a high correlation between time to fall asleep and sleep latency (r = 0.79; *p* < 0.01), a high inverse correlation between participants self-reported effective sleep time and sleep duration (r = −0.72; *p* < 0.01), and a good inverse correlation between effective sleep time and overall PSQI score (r = −0.51; *p* < 0.01). The detail of all correlations is reported in Table 5.

## 4. Discussion

Concerning the main objective of the study, to determine sleep quality in Chilean male professional soccer players, the Pittsburgh questionnaire analysis [42] yielded a value of 4.75 ± 2.29; this value is considered a good sleep quality [47]. In this sense, Swinbourne et al. [49] evaluated sleep quality in professional athletes; these researchers reported a 5.9 ± 2.6 in the Pittsburgh questionnaire and concluded that more than 50% of the evaluated athletes had poor sleep quality (≥5.0 points). In parallel, Khalladi et al. [50] evaluated the sleep characteristics of professional soccer players in the Qatar Stars League. The researchers reported a 68.5% prevalence of players with poor sleep quality (≥5.0 points), concluding that professional soccer players should be more aware of the importance of good sleep quality. It appears that the existing evidence on sleep quality in performance athletes shows worrying indicators [49], even more so in professional soccer players [50]. However, both the overall analysis and the present study showed good sleep quality in Chilean male professional soccer players when comparing the different professional soccer clubs (4.75 ± 2.29). In fact, there were no significant differences between the clubs evaluated (*p* > 0.05), and only one club exceeded five points in the Pittsburgh questionnaire [42].

A vital background to analyze is the hours of sleep reported by the present evaluated clubs (7.27 ± 0.92 h); this period is within the National Sleep Foundation’s recommendations for young people (7–9 h) [10]. Apparently, without considering sleep quality, evidence shows that the amount of sleep hours reported by professional athletes is within the recommendations described above [31,49,50]. An example of this is the data reported by Swinbourne et al. [49]; the researchers evaluated the hours of sleep of 175 professional athletes from various disciplines, reporting an average of 7.9 ± 1.3 h of sleep per day. Likewise, Khalladi et al. [50] reported an average value of over 7.5 h of sleep in professional soccer players, while Caia et al. [31] reported that professional rugby players sleep an average of 7.28 ± 0.8 h per day. However, although evidence shows that elite athletes sleep the recommended number of hours [10], it has also been documented that athletes have sleep problems, identifying three factors that could alter sleep: (a) training, (b) travel, and (c) competition [14]. Consequently, correct sleep hygiene could guarantee positive changes in sleep behavior and a more restful rest [31], which could impact better performance in training and/or competition.

In the present study, when comparing the number of hours of sleep between male professional soccer players who scored ≤5.0 to those who scored over five points on the Pittsburgh questionnaire [42], there were no significant differences between these two groups of soccer players (*p* > 0.05). However, evidence shows a significant difference—between those who score above and below five points—concerning the number of sleeping hours [50]. Despite this, there is still a lack of evidence to ensure that a score above five is associated with sleep outside the National Sleep Foundation’s recommendations (7–9 h) [10]. When, the same comparison was made in the time to fall asleep, a significant difference was found between scores ≤5.0 and above 5 (*p* < 0.001). In this sense, Gupta et al. [14] developed a study that aimed to profile the objective and experienced sleep characteristics among elite athletes. They concluded that cognitive arousal before sleep appears as one of the mechanisms responsible for a longer time to fall asleep [14]; therefore, proper sleep hygiene education may lead to positive sleep behavior changes, which is likely to be beneficial in achieving peak athletic performance [31]. In the present study, regarding the overall PSQI score [42] and the comparison between soccer players with scores ≤5.0 and above five, no significant differences were found between the two groups (*p* > 0.05). However, a previous study showed significant differences in this index when purchasing participants with scores ≤5.0 and over five [50]. Despite this evidence, finding significant differences in these variables does not ensure causality since the results may be conditioned by sample size, group distribution, range, mean values, and standard deviations.

In parallel, a good inverse correlation between effective sleep time and the overall PSQI score was evident (r = −0.51; *p* < 0.01). However, due to the limited evidence relating sleep quality to athletic performance [28], it would be risky to attribute poor athletic performance to sleep deprivation and poor sleep quality. In this regard, Oliver et al. [51] evaluated whether a night of sleep deprivation impaired performance in a treadmill test. The researchers reported decreased performance with limited effect on running pace, and on cardiorespiratory and thermoregulatory function, but no effect on exercise perception. Similarly, Poussel et al. [52] evaluated the relationship between sleep strategies and performance during the 2013 North-Face Ultra-Trail du Mont-Blanc. At the end of the investigation, it was reported that runners who adopted a sleep management strategy based on more sleep time before the race completed the race faster (*p* = 0.02). Furthermore, the researchers reported that most finishers seemed to be aware of the importance of developing sleep management strategies. Contrarily, Blumert et al. [53] compared the effects of 24 h of sleep deprivation on weightlifting performance, concluding that 24 h of sleep deprivation does not affect weightlifting performance. Based on the evidence, when analyzing the effect of sleep deprivation on sports performance, there is a marked difference between strength and endurance sports. Apparently, in the latter sports discipline, sleep deprivation significantly affects sports performance [51,52]. A separate analysis is needed for team sports since most research is focused on the description of sleep quality of athletes [31,49,50] and not on an association between good sleep quality and sports performance [25]. An example of this is the study by Mah et al. [25] who evaluated the effect of sleep length on reaction time, mood, and daytime sleepiness in college basketball players, concluding that optimal sleep is likely to be beneficial for peak athletic performance. Despite this, it is risky to claim that good sleep quality guarantees an athletic outcome in team sports.

Another critical component to consider is the high percentage of male soccer players who reported difficulty falling asleep at some point during the last month (>63%). This difficulty in falling asleep may be directly related to the sleep-wake cycle [54]; in this sense, it has been shown that an instantaneous interruption of sleep as a result of a response to a biological or physiological signal [55] can affect the circadian rhythm of athletes [56]. The latter corresponds to daily cycles that regulate the physiological functions of the organism [57]. Its alteration may contribute to an increased risk of diseases [58] and a decrease in sports performance [56]. Different studies have shown that circadian rhythms cause variations at the hormonal level, in gene expression and body temperature [57,59], directly influencing different components relevant to sports performance such as muscular strength [60], flexibility [59], aerobic endurance [61], and anaerobic power [62]. The sleep-wake cycle is one of the most relevant circadian rhythms for athletes [54], indicating daily rest time [57]. This cycle can be negatively altered due to national or international travel [63], generating a decrease in sports performance due to fatigue and/or jet lag [64]. On the other hand, a score over five in the PSQI could indicate an altered circadian cycle [11]. Since the alteration of the circadian cycle could affect athletes’ physical recovery [56], all the evaluated players should pay attention to it (especially players from Universidad de Chile, who as a whole obtained a score over five in the Pittsburgh questionnaire [42]). This alteration may develop health problems [58] and a decrease in sports performance [14,56].

There are several strategies to improve sleep quality of athletes. As a first measure is the use of electronic devices. An example of these are smartwatches; these devices allow monitoring and evaluating sleep quality efficiently and reliably [65,66]. Another strategy adopted by some athletes to improve sleep quality, and thus sports performance, is the increase of sleep time some nights before the competition [52]. Moreover, in the case of experiencing an acute period of sleep loss, it is suggested to focus more on psychological aspects (motivation) than on the sport’s physiology [53]. Finally, the data obtained reinforces the need to educate professional soccer players and the multidisciplinary team on correct sleep hygiene [29,30,31].

### Limitations

The results of our research show good sleep quality in Chilean male professional soccer players. However, the study did not have a control group to contrast the results with. There was only a qualitative comparison to the recommended values for good sleep quality and its analysis.

As a suggestion for future research and considering the specificity of the group evaluated, we recommend including stress and anxiety evaluations since these factors are mentioned as critical influencers on athletes’ sleep quality and performance.

## 5. Conclusions

According to the results obtained, Chilean male professional soccer players have a good sleep quality. However, the high values of “sleep latency” and “sleep disturbances” are indicators that should be worked on by the multidisciplinary team of each professional club, generating strategies to improve sleep hygiene, encouraging good sleep, and efficient ways of falling asleep.

## 6. Practical Applications

Multidisciplinary teams working with elite athletes must cover all variables associated with sports performance. For this reason, assessing and improving sleep quality is fundamental to ensure proper recovery between training sessions. Consequently, an easy way to monitor sleep quality is through the PSQI. However, to obtain high reliability rates in the application of this test, athletes must be familiarized with it. In this sense, and to increase the reliability of the test, we recommend that multidisciplinary teams regularly apply it to athletes. Finally, in assessing poor sleep quality, we suggest applying sleep hygiene programs to athletes, including a personalized study of the circadian cycle.

## Figures and Tables

**Table 1:** Time and effectiveness of sleep in Chilean male professional soccer players.

	Unión Española (*n* = 20)	Deportes Antofagasta (*n* = 18)	Palestino (*n* = 32)	Universidad de Chile (*n* = 24)	All (*n* = 94)
	Mean ± SD	Mean ± SD	Mean ± SD	Mean ± SD	Mean ± SD
Time to fall asleep (min)	23.65 ± 26.0	32.55 ± 34.4	23.5 ± 21.2	29.75 ± 30.8	26.86 ± 26.9
Effective sleeping time (hours)	7.40 ± 1.41	7.58 ± 0.69	7.24 ± 0.74	7.00 ± 0.72	7.27 ± 0.92
Bedtime (hours) *	8.55 ± 1.13	8.66 ± 1.03	8.08 ± 0.79	7.72 ± 0.89	8.20 ± 1.00

SD: standard deviation; min: minutes; *: *p* < 0.05 between all groups.

**Table 2:** PSQI in Chilean male professional soccer players.

	Unión Española (*n* = 20)	Deportes Antofagasta (*n* = 18)	Palestino (*n* = 32)	Universidad de Chile (*n* = 24)	All (*n* = 94)
	Mean ± SD	Mean ± SD	Mean ± SD	Mean ± SD	Mean ± SD
Subjective sleep quality	0.85 ± 0.67	0.88 ± 0.75	0.87 ± 0.55	1.08 ± 0.71	0.92 ± 0.66
Sleep latency	1.15 ± 0.87	1.16 ± 0.98	1.12 ± 0.97	1.41 ± 0.92	1.21 ± 0.93
Sleep duration	0.35 ± 0.67	0.11 ± 0.32	0.18 ± 0.39	0.37 ± 0.49	0.25 ± 0.48
Habitual sleep efficiency	0.65 ± 0.67	0.50 ± 0.61	0.34 ± 0.60	0.20 ± 0.41	0.40 ± 0.59
Sleep disturbances	1.10 ± 0.30	1.27 ± 0.46	1.18 ± 0.39	1.04 ± 0.46	1.14 ± 0.41
Use of sleeping medication	0.15 ± 0.36	0.05 ± 0.23	0.25 ± 0.56	0.29 ± 0.62	0.20 ± 0.49
Daytime disfunction	0.55 ± 0.60	0.44 ± 0.51	0.68 ± 0.53	0.66 ± 0.70	0.60 ± 0.59
Global score	4.80 ± 2.44	4.44 ± 2.30	4.65 ± 2.25	5.08 ± 2.32	4.75 ± 2.29

PSQI: Pittsburgh Sleep Quality Index; SD: Standard deviation.

**Table 3:** Comparison between male professional soccer players with scores ≤ 5.0 and above five on the Pittsburgh questionnaire.

	≤5.0 (*n* = 64)	>5.0 (*n* = 30)	F	*t*-test	Cohen’s d
	Mean ± SD	Mean ± SD		*p* Value	ES
Time to fall asleep (min)	16.56 ± 14.66	48.83 ± 33.64	36.11	0.003	1.33
Effective sleeping time (hours)	7.53 ± 0.87	6.72 ± 0.78	0.11	0.734	0.98
Bedtime (hours) *	8.26 ± 1.02	8.07 ± 0.94	0.03	0.856	0.19
Subjective sleep quality	0.75 ± 0.53	1.30 ± 0.74	4.16	0.044	0.85
Sleep latency	0.85 ± 0.75	1.96 ± 0.85	0.26	0.605	1.38
Sleep duration	0.09 ± 0.29	0.60 ± 0.62	53.13	0.000	1.10
Habitual sleep efficiency	0.21 ± 0.41	0.80 ± 0.71	14.19	0.000	1.02
Sleep disturbances	1.06 ± 0.35	1.33 ± 0.47	20.82	0.000	0.65
Use of sleeping medication	0.07 ± 0.27	0.46 ± 0.73	60.23	0.000	0.77
Daytime disfunction	0.46 ± 0.56	0.90 ± 0.54	11.93	0.001	0.77
Global score	3.53 ± 1.30	7.36 ± 1.67	1.08	0.300	2.57

SD: standard deviation; min: minutes; ES: effect size; *: *p* < 0.05 between ≤ 5.0 and > 5.0 group.

**Table 4:** Detail of responses to question 5 of the PSQI questionnaire.

Questions	Ranking			
	Has Not Occurred	<1 Time Per Week	1 to 2 Times Per Week	3 or More Times Per Week
Cannot get to sleep within 30 min (%)	37.2	33.0	25.5	4.3
Wake up in the middle of the night or early morning (%)	21.3	36.2	25.5	17.0
Have to get up to use the bathroom (%)	27.7	28.7	25.5	18.1
Cannot breathe comfortably (%)	93.6	2.1	4.3	0.0
Cough or snore loudly (%)	74.5	20.2	3.2	2.1
Feel too cold (%)	66.0	20.2	12.8	1.0
Feel too hot (%)	24.5	26.6	26.6	22.3
Had bad dreams (%)	67.0	25.5	7.5	0.0
Have pain (%)	71.3	20.2	6.4	2.1

PSQI: Pittsburgh Sleep Quality Index.

**Table 5:** Correlations between time and effectiveness of sleep with the PSQI components in Chilean male professional soccer players.

		Time to Fall Asleep	Effective Sleeping Time	Bedtime	C1	C2	C3	C4	C5	C6	C7	Global
Time to fall asleep	r	1.00	−0.31 **	0.02	0.26 **	0.79 **	0.21*	0.46 **	0.01	0.33 **	0.07	0.66 **
	Sig. bil.		0.002	0.846	0.010	0.000	0.037	0.000	0.883	0.001	0.477	0.000
Effective sleeping time	r		1.00	0.67 **	−0.39 **	−0.27 **	−0.72 **	−0.33 **	−0.01	0.09	−0.29 **	−0.51 **
	Sig. bil.			0.000	0.000	0.008	0.000	0.001	0.912	0.347	0.004	0.000
Bedtime	r			1.00	−0.18	−0.01	−0.47 **	0.37 **	0.16	0.20 *	−0.18	−0.03
	Sig. bil.				0.080	0.884	0.000	0.000	0.124	0.047	0.071	0.731
C1	r				1.00	0.23 *	0.39 **	0.21 *	0.27 **	−0.05	0.22 *	0.62 **
	Sig. bil.					0.023	0.000	0.037	0.007	0.620	0.027	0.000
C2	r					1.00	0.21 *	0.30 **	0.05	0.25 *	0.05	0.67 **
	Sig. bil.						0.042	0.003	0.592	0.014	0.593	0.000
C3	r						1.00	0.31 **	0.02	0.00	0.16	0.54 **
	Sig. bil.							0.002	0.827	0.950	0.108	0.000
C4	r							1.00	0.27 **	0.19	0.06	0.62 **
	Sig. bil.								0.007	0.062	0.565	0.000
C5	r								1.00	0.00	0.19	0.41 **
	Sig. bil.									0.932	0.055	0.000
C6	r									1.00	0.05	0.37 **
	Sig. bil.										0.606	0.000
C7	r										1.00	0.44 **
	Sig. bil.											0.000
Global	r											1.00
	Sig. bil.											

PSQI: Pittsburgh Sleep Quality Index; C1: subjective sleep quality; C2: sleep latency; C3: sleep duration; C4: sleep efficiency; C5: sleep disturbances; C6: use of sleeping medication; C7: daytime disfunction; min: minutes; r: Pearson’s correlation index; Sig. bil: significance (bilateral); *: alpha < 0.05 (bilateral); **: alfa < 0.01 (bilateral).

## Data Availability

The data base of the study can be downloaded from the following link: https://figshare.com/articles/dataset/Basedate/14256491 (accessed on 29 April 2021).
